# Pharmacological Chaperone Design for Reducing Risk Factor of Parkinson's Disease from Traditional Chinese Medicine

**DOI:** 10.1155/2014/830490

**Published:** 2014-01-19

**Authors:** Hung-Jin Huang, Cheng-Chun Lee, Calvin Yu-Chian Chen

**Affiliations:** ^1^Department of Chinese Pharmaceutical Sciences and Chinese Medicine Resources, College of Pharmacy, China Medical University, Taichung 40402, Taiwan; ^2^School of Medicine, College of Medicine, China Medical University, Taichung 40402, Taiwan; ^3^Department of Biotechnology, Asia University, Taichung 41354, Taiwan; ^4^China Medical University Beigang Hospital, Yunlin 65152, Taiwan; ^5^Computational and Systems Biology, Massachusetts Institute of Technology, Cambridge, MA 02139, USA

## Abstract

Dysfunction of **β**-glucocerebrosidase (GCase) has no hydrolytic activity in patients of Gaucher's disease and increasing the risk factor for Parkinson's disease occurrence. Pharmacological chaperone design has been used to treat with misfolded protein in related disease, which utilized a small compound to cause protein folding correctly. This study employed the world largest traditional Chinese medicine (TCM) database for searching for potential lead compound as pharmacological chaperone, and we also performed molecular dynamics (MD) simulations to observe the stability of binding conformation between ligands and active site of GCase structure. The docking results from database screening show that N-methylmescaline and shihunine have high binding ability to GCase than tetrahydroxyazepanes. From MD simulation analysis, tetrahydroxyazepanes displayed high opportunity of ligand migration instead of our TCM candidates, and H-bonds number was decreased in the end of MD snapshot. Our result indicated that binding conformation of N-methylmescaline and shihunine remains stable during MD simulation, demonstrating that the two candidates are suitable for GCase binding and might be potential as pharmacological chaperone for GCase folding correctly.

## 1. Introduction

Gaucher's disease (GD) is caused by mutations in the *GBA* gene encoding *β*-glucocerebrosidase (GCase), which leads to inherited glucocerebrosidase deficiency. Because the mutated GCase has no function of hydrolytic activity, the deficient activity of GCase cannot transport from the endoplasmic reticulum (ER) to lysosomes [1]; this phenomenon impaired intracellular transport and contributes to defects of metabolism in fibroblasts of patients [2]. Subsequently, both glucosylceramide (GluCer) and glucosylsphingosine (GluSph) accumulate in macrophages of various organs [3], such as spleen, liver, lungs, bone marrow, and brain [4]; clinical feature of GD disease reveals deposition of undigested subtracts in lysosome of macrophages. In several clinical cases of Gaucher's disease, symptoms of central nervous system (CNS) disorder existed in patient's brain [5]. GD is the most prevalent lysosomal storage diseases [6]; some lines of evidence; indicated that reducing GCase activity is associated with Parkinson's disease (PD) [7, 8] and the mutation of GCase has high risk factor for PD occurrence [9]. Parkinson's disease (PD) is one of the common CNS diseases of neurodegenerative disorders affecting 2% of older adults after the age of 65 [10], which is the second common neuron degenerated disease after Alzheimer's disease [11, 12]. Pathological hallmark of PD is loss of dopaminergic neuron in the pars compacta of the substantia nigra (SNc) [13, 14]; PD patients display reduction of dopamine levels in striatum and degeneration of the dopamine producing neurons, because of the catalytic rate limiting step of tyrosine hydroxylase in catalyzing the conversion of the amino acid tyrosine to L-3,4-dihydroxyphenylalanine (L-DOPA). L-DOPA is a precursor for important neurotransmitters synthesis, including dopamine, noradrenaline, and adrenaline. PD disease can be considered as a tyrosine hydroxylase deficiency [15]. Levodopa (L-DOPA) is wildly used for treatment of PD disease, improving the motor features of PD patients, but there are several side effects remaining in clinical treatment such as fluctuating motor response, dyskinesia, and neuropsychiatric problems [16].

The aim of this study is to focus on pharmacological chaperone (PC) design; the PC therapy utilizes a small molecule to cause misfolded protein structure to fold correctly. Because mutant GCase structure cannot hydrolyse the beta-glucosidic linkage of the GluCer and GluSph, which has high risk factor of PD disease, designing small compounds to promote GCase structure folding correctly is necessary.

Computer-aided drug design (CADD) has been wildly used to design new drugs, which associated with target factors determination [17–21]. TCM compounds regarded as potential leading compounds have been reported in many studies [22–29]. In our study, small molecules from the world largest traditional Chinese medicine (TCM) database [30] were used to search potential compounds with high affinity in GCase active site, and we further utilized molecular dynamics (MD) simulation to observe the stability between protein and ligands for binding assay. Synthetic drug often has sideeffect in clinical treatments; our results could provide nature product as drug candidate, which is more safer and reduce adverse reactions.

## 2. Materials and Methods

### 2.1. Database Screening

The crystal structure of *β*-glucocerebrosidase (GCase) was downloaded from PDB database (PDB code: 3RIK) [31], which were cleaned up mistakes of the crystal structure by Prepare Protein module of Accelrys Discovery Studio 2.5.5.9350 (DS 2.5) software [32], including inserting missing atoms, modeling missing loops, and removing crystal waters. The pH value of 7.4 is specified to protonate all residues. Total number of 61000 TCM compounds were obtained from the TCM database of Taiwan [30] to dock into GCase binding site for binding analysis. All TCM compounds were accessed by ADMET prediction for drug-like evaluation. We performed Ligand docking using LigandFit of DS 2.5; Monte-Carlo technique was used to generate different ligand conformations and docks into the active site of GCase. CHARMm force field was applied to minimize all docked poses. We selected Smart minimizer as minimization algorithm for ligands minimization; this process contains Steepest Descent and Conjugate Gradient. The Steepest Descent performed 1,000 steps and was followed by Conjugate Gradient.

### 2.2. MD Simulation

The molecular dynamic simulation was performed using GROMACS 4.5.5 package [33] with charmm27 force field. Time step of MD simulation was set as 0.002 ps. We used particle mesh Ewald (PME) method [34] as Coulomb type for treating electrostatics and cut-off distance with 1.4 nm to define van der Waals (VDW) residues. The distance of real space cutoff was set to 1.2 nm in define box; LINCS algorithm was used to restrain the lengths of all bonds. We utilized SwissParam to obtain topology file and parameters of small compounds for GROMACS simulation [35]. TIP3P water model was employed in MD simulations; we also used the concentration of 0.145 M NaCl model in the solvent system; the solvent molecules were randomly replaced by Na and Cl ions. Energy minimization using Steepest Descent algorithm and performed 5,000 cycle steps. Equilibration was performed under position restraints for 100 ps. Following this step, production simulations was run for all system for 5000 ps; the temperature of all simulation system was set as 310 K. MD conformations were collected every 20 ps for trajectory analysis.

## 3. Results and Discussion

### 3.1. Docking Analysis of Database Screening

**Figure 1:**
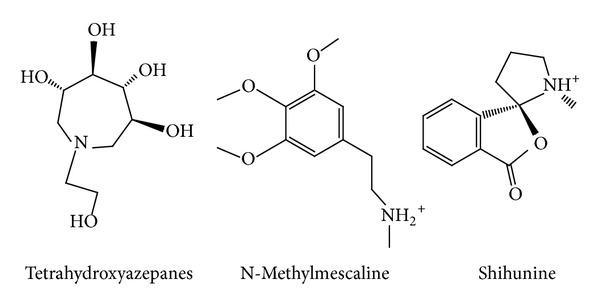
Chemical scaffold of the top 2 TCM candidates (N-methylmescaline and shihunine) and tetrahydroxyazepanes.

**Figure 2:**
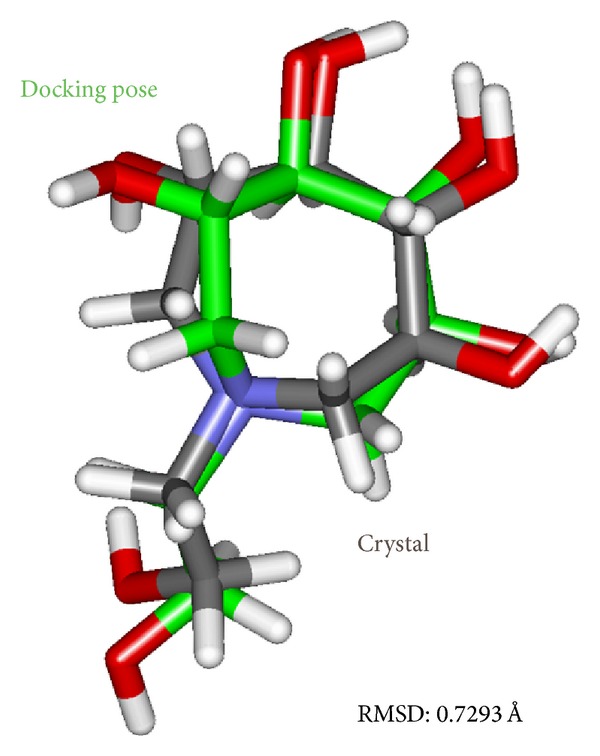
RMSD values of tetrahydroxyazepanes between crystal structure and docking pose.

**Figure 3:**
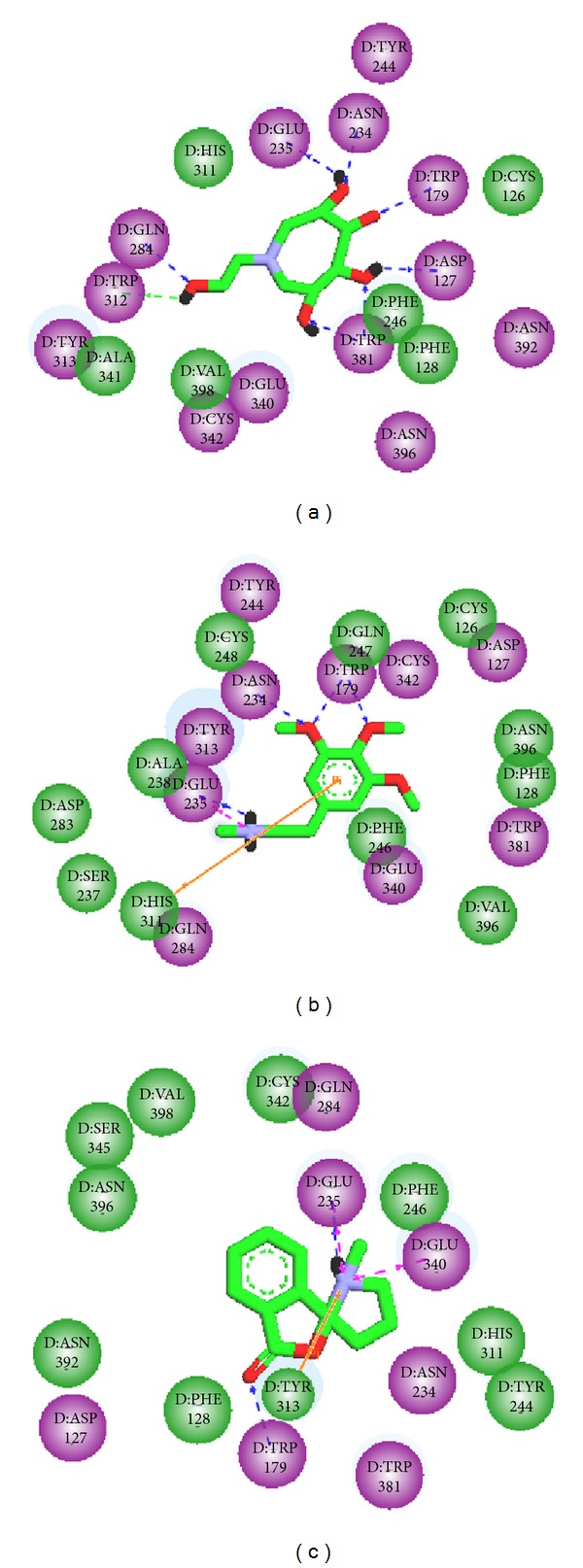
Pi interaction analysis from docking poses of ligands in GBA binding site by Discovery Studio 2.5: (a) tetrahydroxyazepanes, (b) N-methylmescaline, and (c) Shihunine; polar and van der Waals of residues interaction are represented by purple circle and green circle, respectively.

**Figure 4:**
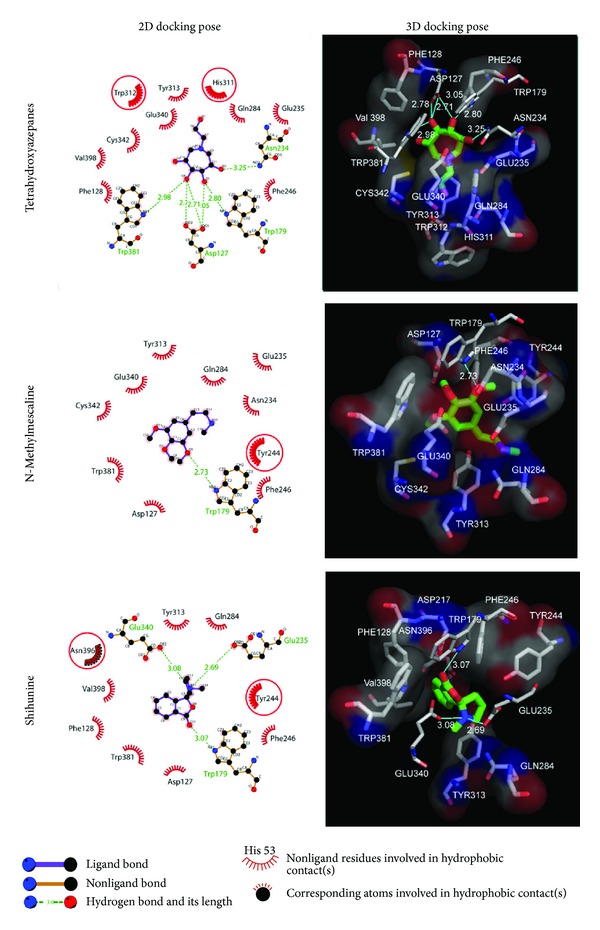
H-bond and hydrophobic interactions analysis of docking poses by Ligplot plus tool; the red circles in 2D plots indicate distinct residues from other docking poses. Residues with solid surface represent hydrophobic interactions in 3D docking poses; H-bonds and ligands are colored in blue and green, respectively.

**Figure 5:**
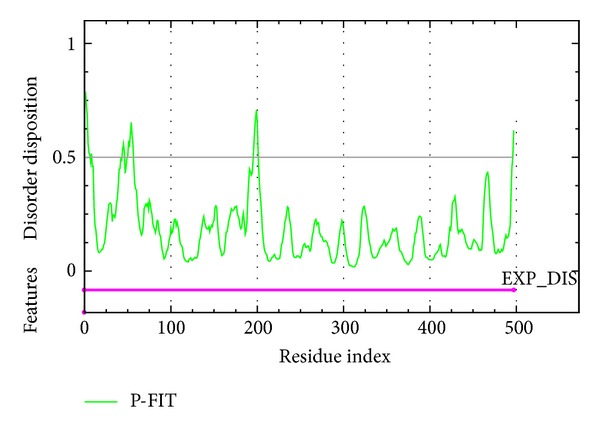
Disorder prediction of GCase by PONDR-FIT, the values above 0.5 in disorder disposition indicate disorder residues.

Docking results of TCM database screening were determined by Dock score and ADMET predictions; top candidates are filtered by comparing with tetrahydroxyazepanes and listed in Table 1. From ADMET prediction, all TCM candidates reveal good or moderate absorption, but tetrahydroxyazepanes have very low absorption, which indicated that our candidates have well movement into the bloodstream. For CYP2D6 and hepatotoxicity, the values in both candidates and control are less than 0.5, which indicated that they have no toxicity to CYP2D6 and no hepatotoxicity in liver. For solubility evaluation, optimal drug-like compounds are present in the top three candidates with 4 values of solubility, and combining with BBB level analysis, N-methylmescaline and shihunine have the highest penetration in blood brain barrier; tetrahydroxyazepanes have too much solubility and no penetration in BBB level. Based on solubility and BBB level, N-methylmescaline and shihunine may be potential lead compounds for CNS diseases, and the dock score is better than control; hence, we selected the top two candidates for further analysis in MD simulation. Chemical scaffolds of the two candidates and control are displayed in Figure 1. The structure of tetrahydroxyazepanes is taken from crystal structure, which has good binding affinity with GCase. N-methylmescaline comes from *Alhagi pseudalhagi* [36], and Shihunine is available in *Dendrobium loddigesii* [37]. To compare docked pose of tetrahydroxyazepanes with X-ray crystal structure by RMSD value of heavy atoms calculation (Figure 2), the two scaffolds are very close to RMSD value of 0.7293 Å, indicating that the predicted binding poses from the LigandFit are matched with experimental poses; all of the compounds from TCM database are according to the binding pose of crystal tetrahydroxyazepanes for binding poses generation. For docking poses, polar and van der Waals (VDW) interactions between residues of GCase and ligand are shown in Figure 3. We found that Glu284, Glu235, Asp127, Trp179, Trp381, Asn234, and Glu340 displayed polar forces among all docking poses, indicating that N-methylmescaline and shihunine have similar binding residues with tetrahydroxyazepanes in GCase active site. For residues of Asn396 and Asn392, which reveals polar interactions with Tetrahydroxyazepanes (Figure 3(a)), but displaying VDW force for N-methylmescaline binding (Figure 3(b)). Asn396 forms VDW interaction for Shihunine interacting, but Asn392 is not shown in Figure 3(c) and has no interactions with the compound. Interestingly, pi interactions are displaced in the 2D diagram of N-methylmescaline and shihunine, which interacts with residue His311 and Tyr313, respectively (Figures 3(b) and 3(c)), but no pi interactions are found in docking pose of tetrahydroxyazepanes (Figure 3(a)). We also utilized Ligplot plus [38] to access hydrophobic interactions between compounds and residues; the distinct interaction of residues is displaced by red circle (Figure 4). Comparing with hydrophobic residues for all docking poses, there are few amino acids are difference between the three docked compounds, suggesting that our candidates have similar hydrophobic interactions with the control set. We further analyze the disorder region of GCase protein; residue indexes of 44–56 and 196–201 are disorder region (Figure 5); all of the binding residues of GCase are not located in disorder region, suggesting that residues of binding site have stable folded structure. The prediction of disorder structure shows that interaction between ligands and residues is not effected by protein structure of GCase [39, 40].

### 3.2. Molecular Dynamics Simulation Analysis

**Figure 6:**
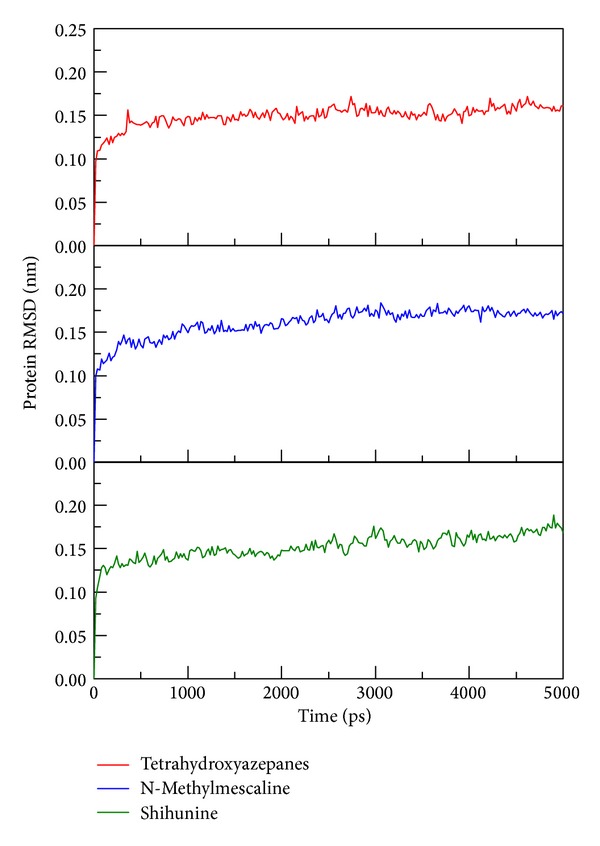
RMSD values of protein structures of the three complexes with docked ligand: tetrahydroxyazepanes, N-methylmescaline, and shihunine among 5000 ps simulation.

**Figure 7:**
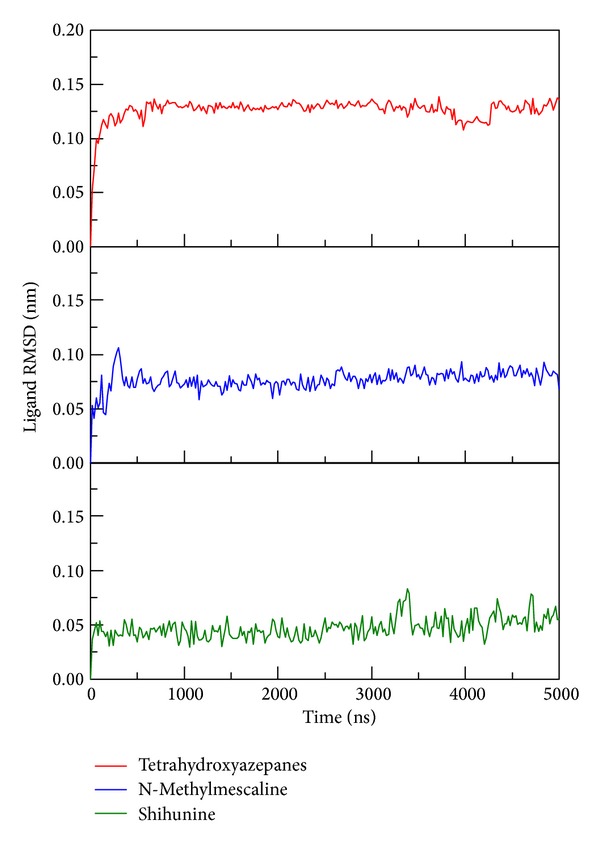
RMSD values of three compounds: tetrahydroxyazepanes, N-methylmescaline, and shihunine in complexes among 5000 ps simulation.

**Figure 8:**
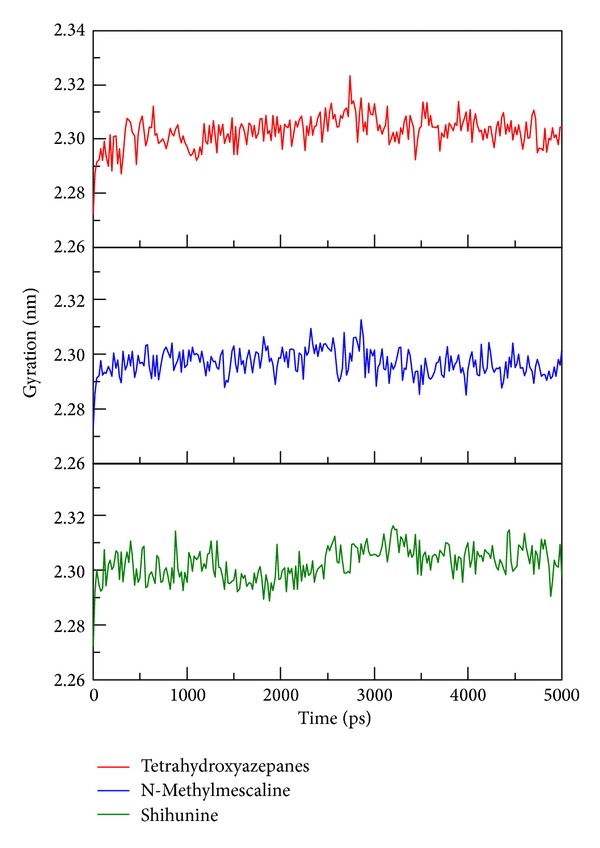
Protein gyration values of the three complexes with docked ligand: tetrahydroxyazepanes, N-methylmescaline, and shihunine among 5000 ps simulation.

**Figure 9:**
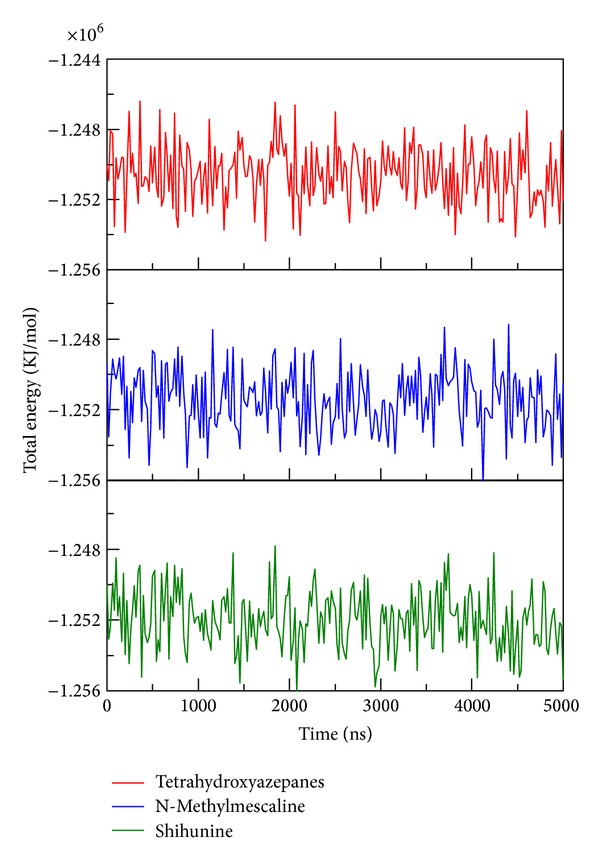
Total energy of three complexes with docked ligand: tetrahydroxyazepanes, N-methylmescaline, and shihunine among 5000 ps simulation.

To determine stability of conformations among MD simulation, we employed root mean square deviation (RMSD), radius of protein gyration, and total energy to analyze the deviation of all complexes with docked ligand. The RMSD values of protein structure were used to verify stability among MD simulation.  Figure 6 shows all values within the range from 0.15 to 0.18 nm, which indicate that all protein structures from protein-ligand complexes are stable during 5000 ps simulation and the 5000 ps simulation time is enough for decreasing the fluctuation of all complexes. For ligand RMSD (Figure 7), it is obvious that the conformation of tetrahydroxyazepanes displays high degree of difference during dynamic simulation; the value of ligand RMAD increased to 0.15 nm from 500 ps to 5000 ps. The ligand RMSD of TCM candidates revealed slight deviation; the increased values of N-methylmescaline and shihunine are 0.08 and 0.05, respectively. Radius of protein gyration (Rg) was used to observe the compactness of protein structure (Figure 8); the stability of the gyration plot is similar to protein RMSD; gyration values of the three complexes remain at 2.30 nm for all simulation times, which show that all of the protein complexes are more compact among dynamic simulation. Total energy shows that N-methylmescaline and shihunine have lower energy values (below −1.252 × 10^6^ KJ/mol) than tetrahydroxyazepanes (above −1.252 × 10^6^ KJ/mol) in GCase complexes (Figure 9), suggesting that both of TCM compounds are more stable in protein structure. Because the plots of ligand RMSD and total energy display significant change, observing the variation of tetrahydroxyazepanes between initial pose and dynamic pose for comparing with TCM candidates is necessary.

### 3.3. Comparison between Initial and Dynamic Conformations

**Figure 10:**
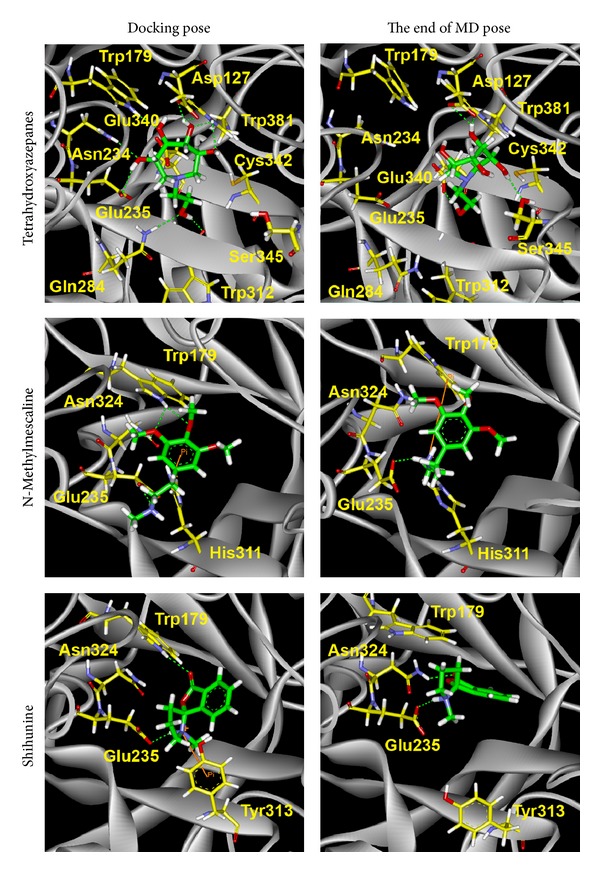
Docking poses compare to the end of MD conformation; Pi stack interactions and hydrogen bonds are colored in origin and green, respectively.

**Figure 11:**
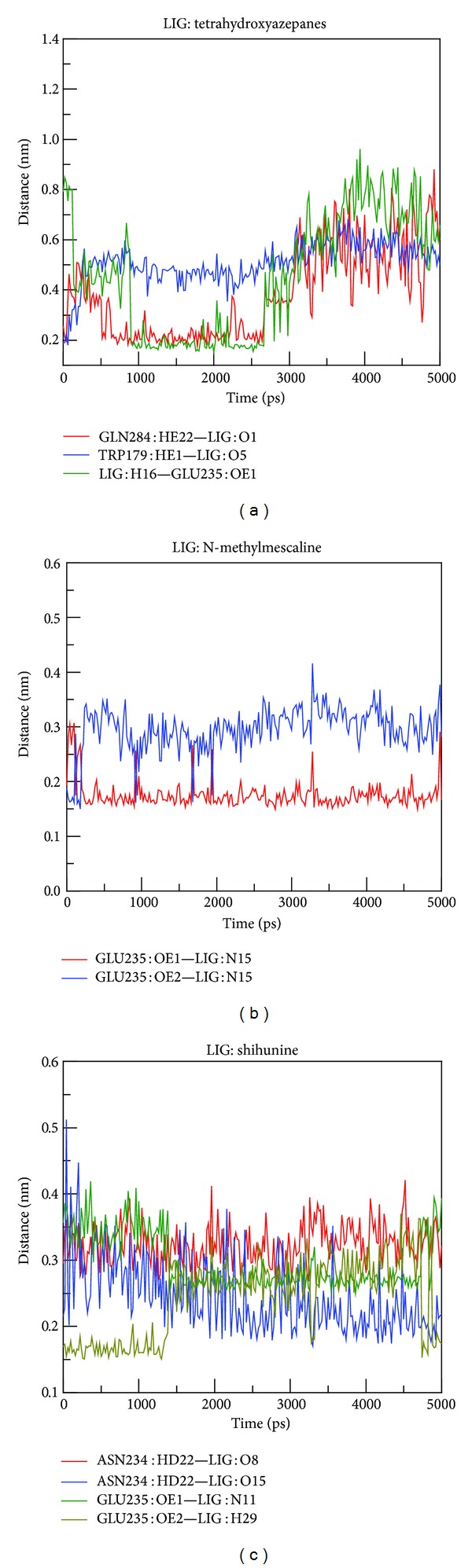
H-bond distance between residues and ligands among all simulation times: (a) tetrahydroxyazepanes, (b) N-methylmescaline, and (c) shihunine.

In order to observe conformation variation between docking pose and the end of MD pose, snapshots of protein-ligand complexes were listed in Figure 10. Docking pose of tetrahydroxyazepanes shows that H-bonds were formed with eight amino acids (Asp127, Trp179, Glu340, Asn234, Glu235, Gln284, Trp312, and Trp381), but the final MD conformation shows that lots of H-bonds are disappeared, the H-bond is only displayed on Asp127, Ser345, and Glu340 in the active site, and this phenomenon refers to the high degree value in ligand RMSD plot. Docking pose of N-methylmescaline has pi-cation interaction with His311 and forms H-bond with Trp179, Asn234, and Glu235. Interestingly, the pi-cation changes to Trp179, and Glu235 still has H-bond, which indicated that N-methylmescaline reveals stable binding affinity in GCase active site after MD. For shihunine snapshots analysis, pi-cation interaction is formed with Tyr313 and generated H-bond with Trp179 and Glu235, but in the end of MD conformation H-bonds change from Trp179 to Asn234; Glu235 still keeps the H-bond with ligand. The binding interactions of shihunine in final pose are less than in docking pose; however, the conformation of ligand does not change significantly after MD simulation. The slight increased value of ligand RMSD also supported this finding, which indicated that shihunine could form stable binding pose in GCase active site. We also measured the distance of H-bond during MD simulation; the distance significantly increases to 0.7 nm between tetrahydroxyazepanes and three residues: Gln284, Trp179 and Glu235 (Figure 11(a)); N-methylmescaline and shihunine remain within 0.35 during all simulation time (Figures 11(b) and 11(c)). The variations of H-bond distance are correlated with comparison of snapshots between docking and MD poses.

### 3.4. Migration Assay of Ligands in GCase

**Figure 12:**
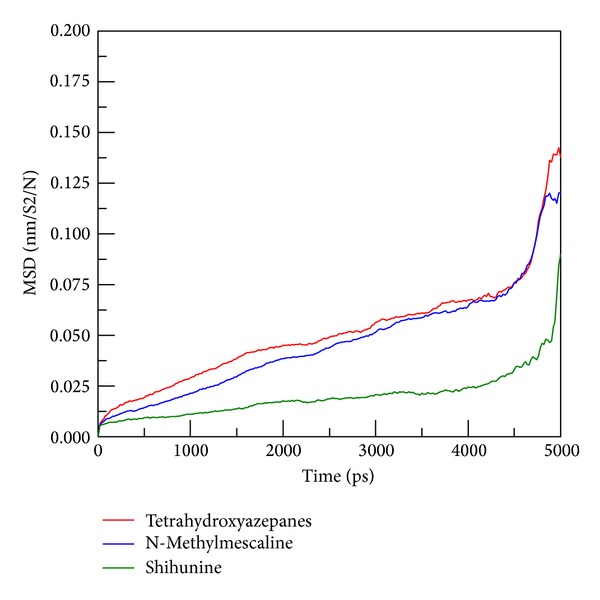
Mean square displacement (MSD) of different ligands during all simulation times; more increased value of MSD indicates higher migration of docked ligand away from initial site.

**Figure 13:**
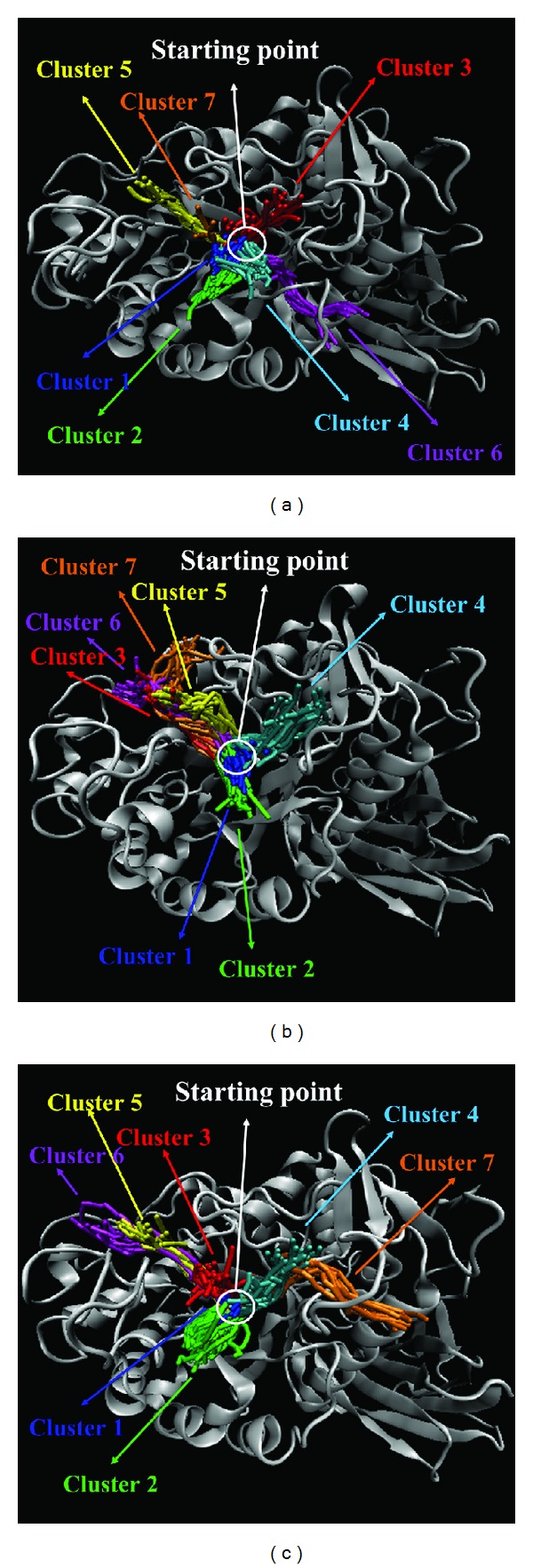
Ligand pathway of (a) tetrahydroxyazepanes, (b) N-methylmescaline, and (c) shihunine in GCase binding site by CAVER 3.0; starting point is the initial place for ligand binding; all generated channels are grouped to seven clusters.

Because the tetrahydroxyazepanes have significant difference between docking and MD poses, we further computed the mean square displacement (MSD) of ligand atoms to analyze the migration of docked ligands among all simulation times. MSD value of tetrahydroxyazepanes are higher than our two candidates (Figure 12). We utilized CAVER 3.0 software [41] to predict the migrated ligand tunnels in GCase; the network of ligand pathway has been used to analyze the binding stability of docked compounds [42]. The seven clusters of ligand channels are shown in Figure 13; it is worthy to note that there are three different directions of clusters for N-methylmescaline and shihunine to migrate from starting point (Figures 13(b) and 13(c)), but tetrahydroxyazepanes generated four different directions of clusters in GCase; the distinct tunnel is cluster 6 (Figure 13(a)); this result reveals that tetrahydroxyazepanes have higher opportunity than N-methylmescaline and shihunine to cause ligand to move away from the docked site.

## 4. Conclusion

In ADMET prediction, N-methylmescaline and shihunine have optimal drug-like features and medium blood brain barrier penetration ability; both of the two TCM candidates are more potential lead compounds than tetrahydroxyazepanes in CNS disease treatments [42–52]. For docking analysis, both N-methylmescaline and shihunine have higher Dock score than control, which have pi-cation interactions in docking pose snapshots instead of tetrahydroxyazepanes. All of binding residues reveal folding stable from protein disorder prediction, which indicated that the docked ligand have no effect by disorder region to avoid unstable binding. We further analyze the conformational changes of protein-ligand complexes after MD simulation; tetrahydroxyazepanes displayed significant change on ligand RMSD plot analysis, but N-methylmescaline and shihunine were stable during MD simulation. Besides, the end of MD snapshots showed that tetrahydroxyazepanes decreased H-bond number after MD simulation; the end of MD poses of our candidates is similar to docking poses, suggesting that N-methylmescaline and shihunine have high affinity with GCase among all MD simulation. For ligand migration analysis, tetrahydroxyazepanes have the highest distance of movement in MSD evaluation. In addition, the ligand paths from channel prediction reveal more directions from initial binding position; this result is correlated with the comparison of snapshots. Our result indicated that N-methylmescaline and shihunine might be potential lead compounds to design pharmacological chaperone for GCase folding correctly and reducing the risk factor for PD occurrence.

## Figures and Tables

**Table 1:** Results of TCM database screening from LigandFit Docking protocol; all candidates are evaluated by ADMET prediction.

Name	Dock score	ADMET prediction				
		Absorption^a^	CYP2D6^b^	Hepatotoxicity^c^	Solubility^d^	BBB level^e^
N-Methylmescaline	63.879	0	0	0	4	2
Shihunine	48.998	0	0	0	4	2
Selagine	60.932	0	0	0	4	3
Rosmaricine	52.749	0	0	0	3	3
Aucubigenin	48.536	1	0	0	5	3
Genipic acid	48.287	1	0	0	5	3
Ascorbic acid	53.255	1	0	0	5	4
Tetrahydroxyazepanes*	48.207	3	0	0	5	4

*Control

^a^Absorption: good absorption: 0; moderate absorption: 1; low absorption: 2; very low absorption: 3; ^b^CYP2D6: noninhibitor < 0.5; inhibitor > 0.5; ^c^Hepatotoxicity: nontoxic < 0.5; toxic > 0.5; ^d^solubility: good druglikeness: 3; optimal druglikeness: 4; too solubility: 5; ^e^blood brain barrier (BBB) level: medium penetration: 2; low penetration: 3; undefined penetration: 4.

## References

[1] Zimmer KP, le Coutre P, Aerts HM (1999). Intracellular transport of acid beta-glucosidase and lysosome-associated membrane proteins is affected in Gaucher's disease (G202R mutation). *Journal of Pathology*.

[2] Jonsson LMV, Murray GJ, Sorrell SH (1987). Biosynthesis and maturation of glucocerebrosidase in Gaucher fibroblasts. *European Journal of Biochemistry*.

[3] Dasgupta N, Xu YH, Oh S (2013). Gaucher disease: transcriptome analyses using microarray or mrna sequencing in a gba1 mutant mouse model treated with velaglucerase alfa or imiglucerase. *PLoS One*.

[4] Cassinerio E, Graziadei G, Poggiali E (2013). Gaucher disease: a diagnostic challenge for internists. *European Journal of Internal Medicine*.

[5] Lieberman RL (2011). A guided tour of the structural biology of gaucher disease: acid-beta-glucosidase and saposin C. *Enzyme Research*.

[6] Schonemann W, Gallienne E, Ikeda-Obatake K (2013). Glucosylceramide mimics: highly potent GCase inhibitors and selective pharmacological chaperones for mutations associated with types 1 and 2 Gaucher disease. *ChemMedChem*.

[7] Rosenbaum H, Aharon-Peretz J, Brenner B (2013). Hypercoagulability, parkinsonism, and Gaucher disease. *Seminars in Thrombosis and Hemostasis*.

[8] Neudorfer O, Giladi N, Elstein D (1996). Occurrence of Parkinson’s syndrome in type I Gaucher disease. *QJM*.

[9] Li Y, Sekine T, Funayama M (2013). Clinicogenetic study of GBA mutations in patients with familial Parkinson's disease. *Neurobiology of Aging*.

[10] Federoff M, Jimenez-Rolando B, Nalls MA, Singleton AB (2012). A large study reveals no association between APOE and Parkinson’s disease. *Neurobiology of Disease*.

[11] Wirdefeldt K, Adami H-O, Cole P, Trichopoulos D, Mandel J (2011). Epidemiology and etiology of Parkinson’s disease: a review of the evidence. *European Journal of Epidemiology*.

[12] Nussbaum RL, Ellis CE (2003). Alzheimer’s disease and Parkinson’s disease. *The New England Journal of Medicine*.

[13] Prensa L, Cossette M, Parent A (2000). Dopaminergic innervation of human basal ganglia. *Journal of Chemical Neuroanatomy*.

[14] Roede JR, Uppal K, Park Y (2013). Serum metabolomics of slow vs. rapid motor progression Parkinson’s disease: a Pilot Study. *PLoS One*.

[15] Haavik J, Toska K (1998). Tyrosine hydroxylase and Parkinson’s disease. *Molecular Neurobiology*.

[16] Olanow CW, Jankovic J (2005). Neuroprotective therapy in Parkinson’s disease and motor complications: a search for a pathogenesis-targeted, disease-modifying strategy. *Movement Disorders*.

[17] Lee CC, Tsai CH, Wan L (2013). Increased incidence of Parkinsonism among Chinese with *β*-glucosidase mutation in central Taiwan. *BioMedicine*.

[18] Chou IC, Lin WD, Wang CH (2013). Association analysis between Tourette's syndrome and two dopamine genes (DAT1, DBH) in Taiwanese children. *BioMedicine*.

[19] Wu PL, Lane HY, Tang HS, Tsai GE (2012). Glutamate theory in developing novel pharmacotherapies for obsessive compulsive disorder: focusing on N-methyl-D-aspartate signaling. *BioMedicine*.

[20] Wang CH, Lin WD, Bau DT (2013). Appearance of acanthosis nigricans may precede obesity: an involvement of the insulin/IGF receptor signaling pathway. *BioMedicine*.

[21] Su KP (2012). Inflammation in psychopathology of depression: clinical, biological, and therapeutic implications. *BioMedicine*.

[22] Chen K-C, Yu-Chian Chen C (2011). Stroke prevention by traditional Chinese medicine? A genetic algorithm, support vector machine and molecular dynamics approach. *Soft Matter*.

[23] Tsai T-Y, Chang K-W, Chen CY-C (2011). IScreen: world’s first cloud-computing web server for virtual screening and de novo drug design based on TCM database@Taiwan. *Journal of Computer-Aided Molecular Design*.

[24] Chang S-S, Huang H-J, Chen CY-C (2011). Two birds with one stone? Possible dual-targeting H1N1 inhibitors from traditional Chinese medicine. *PLoS Computational Biology*.

[25] Yang S-C, Chang S-S, Chen H-Y, Chen CY-C (2011). Identification of potent EGFR inhibitors from TCM Database@Taiwan. *PLoS Computational Biology*.

[26] Chen K-C, Chang K-W, Chen H-Y, Chen CY-C (2011). Traditional Chinese medicine, a solution for reducing dual stroke risk factors at once?. *Molecular BioSystems*.

[27] Chen K-C, Sun M-F, Yang S-C (2011). Investigation into potent inflammation inhibitors from traditional chinese medicine. *Chemical Biology and Drug Design*.

[28] Tou WI, Chang SS, Lee CC, Chen CY (2013). Drug design for neuropathic pain regulation from traditional Chinese medicine. *Scientific Reports*.

[29] Hor LI, Chen CL (2013). Cytotoxins of Vibrio vulnificus: functions and roles in pathogenesis. *BioMedicine*.

[30] Chen CY-C (2011). TCM Database@Taiwan: the world’s largest traditional Chinese medicine database for drug screening In Silico. *PLoS ONE*.

[31] Orwig SD, Tan YL, Grimster NP (2011). Binding of 3,4,5,6-tetrahydroxyazepanes to the acid-*β*-glucosidase active site: implications for pharmacological chaperone design for Gaucher disease. *Biochemistry*.

[32] Accelerys (2009). *Discovery Studio Client v2. 5*.

[33] Pronk S, Pall S, Schulz R (2013). GROMACS 4. 5: a high-throughput and highly parallel open source molecular simulation toolkit. *Bioinformatics*.

[34] Cheatham TE, Miller JL, Fox T, Darden TA, Kollman PA (1995). Molecular dynamics simulations on solvated biomolecular systems: the particle mesh Ewald method leads to stable trajectories of DNA, RNA, and proteins. *Journal of the American Chemical Society*.

[35] Zoete V, Cuendet MA, Grosdidier A, Michielin O (2011). SwissParam: a fast force field generation tool for small organic molecules. *Journal of Computational Chemistry*.

[36] Sun W, Sneng J (1998). *Brief Handbook of Natural Active Compounds*.

[37] Li MF, Hirata Y, Xu GJ, Niwa M, Wu HM (1991). Studies on the chemical constituents of Dendrobium loddigesii rolfe. *Acta Pharmaceutica Sinica*.

[38] Laskowski RA, Swindells MB (2011). LigPlot+: multiple ligand-protein interaction diagrams for drug discovery. *Journal of Chemical Information and Modeling*.

[39] Tou WI, Chen CY (2013). May disordered protein cause serious drug side effect?. *Drug Discovery Today*.

[40] Chen CY, Tou WI (2013). How to design a drug for the disordered proteins?. *Drug Discov Today*.

[41] Chovancova E, Pavelka A, Benes P (2012). CAVER 3. 0: a tool for the analysis of transport pathways in dynamic protein structures. *PLOS Computational Biology*.

[42] Chen KC, Chang SS, Huang HJ (2012). Three-in-one agonists for PPAR-alpha, PPAR-gamma, and PPAR-delta from traditional Chinese medicine. *Journal of Biomolecular Structure and Dynamics *.

[43] Liao W-L, Tsai F-J (2013). Personalized medicine: a paradigm shift in healthcare. *BioMedicine*.

[44] Tsai F-J (2011). Biomedicine brings the future nearer. *BioMedicine*.

[45] Tsai F-J (2013). Rare diseases: a mysterious puzzle. *BioMedicine*.

[46] Chou IC, Lin W-D, Wang C-H (2013). Möbius syndrome in a male with XX/XY mosaicism. *BioMedicine*.

[47] Lee C-C, Tsai C-H, Wan L (2013). Increased incidence of Parkinsonism among Chinese with *β*-glucosidase mutation in central Taiwan. *BioMedicine*.

[48] Lin D-Y, Tsai  F-J, Tsai C-H, Huang C-Y (2011). Mechanisms governing the protective effect of 17*β*-estradiol and estrogen receptors against cardiomyocyte injury. *BioMedicine*.

[49] Wang C-H, Lin W-D, Bau D-T (2013). Appearance of acanthosis nigricans may precede obesity: An involvement of the insulin/IGF receptor signaling pathway. *BioMedicine*.

[50] Chang Y-M, Velmurugan BK, Kuo W-W (2013). Inhibitory effect of alpinate Oxyphyllae fructus extracts on Ang II-induced cardiac pathological remodeling-related pathways in H9c2 cardiomyoblast cells. *BioMedicine*.

[51] Chou IC, Lin W-D, Wang  C-H (2013). Association analysis between Tourette's syndrome and two dopamine genes (DAT1, DBH) in Taiwanese children. *BioMedicine*.

[52] Lin W-Y, Liu H-P, Chang J-S (2013). Genetic variations within the PSORS1 region affect Kawasaki disease development and coronary artery aneurysm formation. *BioMedicine*.

